# Exploring Climate Change’s Impact on the Cardiopulmonary Health of Adults Living in the Canton of Valais, Switzerland: Protocol for a Development and Usability Pilot Study

**DOI:** 10.2196/67128

**Published:** 2025-03-25

**Authors:** Omar Portela Dos Santos, Paulo Jorge Pereira Alves, Henk Verloo

**Affiliations:** 1 Department of Nursing Sciences School of Health Sciences HES-SO Valais/Wallis Sion Switzerland; 2 Institute of Health Sciences Universidade Católica Portuguesa Porto Portugal; 3 Service of Old Age Psychiatry Department of Psychiatry Lausanne University Hospital Lausanne Switzerland

**Keywords:** climate change, global warming, emergency department, emergency nursing, sustainable care, ecological medicine, cardiopulmonary, cardio health, Valais, Switzerland, pilot study, study protocol, humanity, air pollution, impact, comorbidities, adults, mixed methods design, feasibility, health promotion, disease prevention, acceptability

## Abstract

**Background:**

Climate change is affecting public health and well-being. In 2016, Swiss emergency departments (EDs) treated 1,722,000 cases, with 4718 daily admissions. In 2023, the ED of Sion Regional Hospital recorded 75,000 consultations. The links between climate change and health are complex, necessitating urgent research on its impact on cardiopulmonary health in Valais, Switzerland. Raising awareness among frontline professionals is crucial for developing health promotion and disease prevention strategies.

**Objective:**

This study explores the preliminary effects of climate change on cardiopulmonary health in Valais and assesses adult patients’ knowledge of its health consequences. Findings will inform adaptations in patient care, health promotion, and disease prevention at Sion Hospital’s ED. The feasibility of patient selection and data collection will also be evaluated.

**Methods:**

Using a convergent, parallel, mixed methods design, data will be collected from September 21, 2024, to September 20, 2025, with a target sample of 60 patients. The quantitative phase will examine patient recruitment feasibility, consultation reasons, and triage levels, correlating them with climate variables (temperature, nitrogen dioxide, particulate matter, sulfur dioxide, and ozone). It will also analyze sociodemographic profiles. The qualitative phase will explore patients’ knowledge of climate change and its potential links to their ED visits. The feasibility and acceptability of the study process will be assessed. The protocol follows the SPIRIT (Standard Protocol Items: Recommendations for Interventional Trials) Extension for Pilot and Feasibility Trials.

**Results:**

Data collection started on September 21, 2024, following the approval by the ethical commission. Data collection will take place over 1 year, until September 20, 2025.

**Conclusions:**

This study will test the feasibility of a larger investigation and examine potential associations between Valais’ changing microclimate and population health. Findings will establish patient profiles and explore their perceptions and knowledge of climate change, informing future health interventions.

**International Registered Report Identifier (IRRID):**

DERR1-10.2196/67128

## Introduction

The planet’s climate crisis is directly damaging human health. Globally, 3.6 billion people live in areas highly sensitive to climate change. Rising temperatures, extreme weather events, air pollution, and the spread of infectious diseases are just some of the major health threats exacerbated by climate change [1]. Climate change has many other direct and indirect effects on human health, including physical and mental disorders. Projections for 2025 estimate that climate change will be responsible for 250,000 additional deaths per year between 2030 and 2050 [2,3]. Worldwide, 9 out of 10 people breathe poor-quality air, and more than 7 million people die every year because of this pollution [4]. Greenhouse gas emissions, particulate matter (PM), nitrogen dioxide (NO_2_), tropospheric ozone (O_3_), and sulfur dioxide (SO_2_) are the pollutants with the most significant impact on human health. There has been a concurrent 57% increase in heatwave episodes since 2010. Heat-related deaths among people >65 years of age have risen by 70% in 2 decades [5].

In recent years, several studies have been conducted on the various components of the relationship between health and climate change in Switzerland, particularly via the Swiss Study on Air Pollution and Lung and Health Diseases in Adults cohort [6]. A review of 22 studies by Cicci et al [7] highlighted positive associations between high temperatures and ischemic heart disease, acute myocardial infarction, the risk of congestive heart failure, and the number of emergency department (ED) consultations. Indeed, increasing numbers of ED admissions [7,8] hospitalizations for respiratory and cardiovascular diseases have been linked to nonoptimal temperatures and exposure to pollutants and unconventional natural gas development, while more cases of decreased lung function or chronic obstructive pulmonary disease have been linked to exposure to SO_2_, NO_2_, and PM_10_ [9-11]. Finally, climate change is leading to global warming, that is, the phenomenon of increasing average air temperatures near the Earth’s surface [12]. This prolongs plant growth and pollination seasons and often increases the overall amount of pollen produced. This phenomenon can lead to increased respiratory allergies, rhinitis, and asthma in sensitive patients with immunoglobulin E–mediated allergic reactions [13,14].

In addition to its impacts on health, climate change can compromise many social determinants of good health, exacerbating the inequalities in morbidity and mortality that particularly threaten vulnerable populations. Indeed, environmental risk factors, such as demographics, geography, biology, health, sociopolitical and socioeconomic status, health system capacity, and overall equity, are responsible for 80% of common illnesses and 25% to 33% of the total disease burden [15]. Vulnerability has 4 primary features: integrity (a person’s sense of soundness), challenge (vulnerability is experienced when there is a perceived challenge to one’s integrity and uncertainty about how to respond to it), capacity for action (the perceived ability to withstand, integrate or cope with the challenge), and multidimensionality (how vulnerability varies from one person to another and from one experience to another) [16,17]. Older adults, infants from the age of 0-1 year, people with chronic diseases, those living in urban environments with low socioeconomic status or experiencing social isolation [5,18], and people living at higher altitudes [7] are all considered to be population groups vulnerable to climate change. Finally, regarding biological sex, Bayentin et al [19] and Gebhard et al [20] found that hospitalizations for ischemic heart diseases and myocardial infarction were higher among younger women than among younger men. Women have a higher core temperature, skin temperature, heart rate, and blood pressure than men, which can lead to decreased heat tolerance. However, men have a 33% higher incidence of stroke and a 41% higher prevalence of stroke than women [7].

EDs are gateways to the health care system. Their mission is to provide immediate specialist care to patients with urgent or life-threatening needs. Despite their heterogeneous profiles, EDs must provide patients with efficient, high-quality care, which the Institute of Medicine defines as the ability of a health service to increase the likelihood of achieving desired health outcomes in line with current professional knowledge [21]. Quality of care is a multidimensional concept that implies safe, responsive, effective, efficient, equitable, and patient-centered care [21]. In Switzerland, the National Ordinance on Quality of Care and Patient Safety sets out how health care facilities, institutions, and health care professionals must be actively committed to ensuring the quality of care and promoting patient safety. Moreover, through their collaboration, patients contribute to achieving the defined objectives of high-quality care and safety.

Ever more attention is being paid to the relationship between the environment and health. Climate change is causing health problems that did not exist before, leading to new health care needs. It is, therefore, essential to explore preliminary associations between climate variables and sociodemographic and health variables. Specifically, the primary outcome of the quantitative phase is the systematic assessment and analysis of the feasibility of patient recruitment, the reasons for their consultation, and their triage level. This first outcome will be correlated with climate variables (maximum and minimum temperature and concentrations of NO_2_, PM, SO_2_, and O_3_) to investigate whether climate variables influence hospital admissions. The secondary outcome is an exploration of the sociodemographic profiles of adult patients consulting at Sion Hospital’s ED. For the qualitative stage, the primary outcome is evaluating the level of acceptability of an interview guide to explore patients’ knowledge about climate change and its potential links with their ED visits, and the secondary outcome is an exploration of patients’ knowledge about climate change. Given the nature of the study, we decided to focus solely on cardiorespiratory comorbidities. As part of a larger study, other comorbidities should be explored, such as metabolic comorbidities (metabolic syndrome; type 2 diabetes, often associated with cardiovascular disorders; hypercholesterolemia; and hyperuricemia or gout), psychiatric comorbidities (depression, anxiety disorder, schizophrenia, and bipolar disorder), musculoskeletal comorbidities (osteoarthritis, fibromyalgia, and osteoporosis), endocrine comorbidities (hypothyroidism and hyperthyroidism), or gastrointestinal comorbidities (celiac disease and inflammatory bowel disease).

Nurses are pivotal in recognizing and addressing the direct health impacts of climate change. They are uniquely positioned to promote overall health with their sensitivity to patients’ vulnerabilities and emerging health needs. This includes identifying how climate change affects health in their daily practice, educating patients and communities about related risks, and fostering environmentally sustainable behaviors [22]. To achieve this, studies must be conducted on climate change and its health consequences. The first step is to assess the feasibility of the methods and procedures for a future large-scale study.

## Methods

### Design

Achieving this development and usability pilot study’s objectives requires a convergent, parallel, mixed methods design. Pilot studies are commonly used in health-related research in disciplines such as nursing and medicine [23], frequently to generate data for sample size calculations. This seems especially relevant when there are no data from previous studies to inform the process [24-26].

### Quantitative Phase

#### Overview

**Figure 1:**
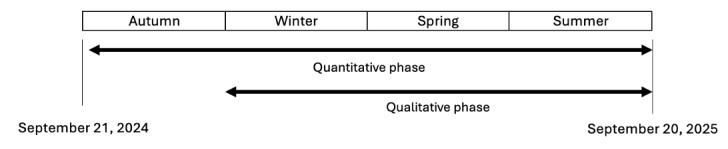
Pilot project’s quantitative and qualitative phases.

Data collection will occur from September 21, 2024, to September 20, 2025 (Figure 1).

The quantitative phase’s objectives are to assess and analyze patient recruitment’s feasibility, the reasons for adult patients’ consultations at Sion’s ED, their triage level, and their sociodemographic profiles. These variables will be analyzed with climate variables (maximum and minimum temperatures and concentrations of NO_2_, PM, SO_2_, and O_3_) to investigate potential associations between hospital admissions and various climate variables. The secondary outcome is an exploration of the sociodemographic profiles of the adult patients consulting at Sion’s ED.

A sample size of 40 to 60 adult patients is planned, that is, 15 per season. Pilot studies are often conducted to generate data for sample size calculations. This seems especially sensible when there are no data from previous studies to inform this process. This is the case in the context of this study. It is the first time such a study has been carried out in the canton of Valais. A general rule of thumb is to take 30 patients or greater to estimate a parameter [24,26]. During the triage process, the triage nurse recruits patients for the quantitative phase. Then, the nurse in charge collects the variables of interest.

#### Inclusion Criteria and Data Collection

We will include all adult patients aged 18 years and older, who consulted at Sion’s ED and have cardiopulmonary comorbidities (ischemic heart disease, hypertensive heart disease, heart arrhythmias, heart failure, chronic obstructive pulmonary disease, asthma, obstructive sleep apnea, pneumonia, pulmonary fibrosis, and pulmonary cancer) sorted into levels 3, 4, or 5 according to the Valais triage scale. According to the Valais Swiss sorting scale, level 3 is considered semiurgent with a maximum response time is 60 minutes. Levels 4 and 5 are nonurgent, with a waiting time of 120 and 180 minutes, respectively (nonurgent). The patient must be able to speak, understand, and read French and sign the informed consent form with full knowledge of the facts. Eligible participants who sign this form but die during or after their ED consultation will be included in the study. The exclusion criteria are patients classified at levels 1 or 2 on the triage scale or who do not possess the capacity for discernment (as diagnosed by an ED physician). A level-1 classification on the Valais triage and severity scale implies a life-threatening condition. The patient’s pathological situation could lead to death or the loss of an organ or a limb if care is not provided immediately. The patient must be transferred to an emergency care unit immediately. A level-2 triage classification requires urgent treatment since the pathological situation is not life-threatening but is susceptible to rapid deterioration. The patient must be transferred to an emergency care unit and assessed by a physician as quickly as possible. We believe it is, therefore, inadvisable to involve patients triaged at levels 1 and 2 in the pilot study as their rapid care is vital. Finally, Sion’s ED does not accept patients under 18 years of age, as they are referred directly to the pediatric ED.

The variables to be analyzed can be divided into two categories: (1) sociodemographic data such as sex, age, place of residence (commune and postal code) and marital status (single, married, divorced, widowed, separated, or in a couple); and (2) medical and clinical data collected during ED visits, such as triage level, reason for the consultation, diagnosis based on the *ICD-10* *(International Statistical Classification of Disease, Tenth Revision*) classification, medical or surgical history, smoking status, and ED readmissions in the last 6 months. All these questions, except for ED readmissions in the last 6 months, will be collected by the nurse in charge during history taking and other exchanges with the patient. In this way, the data collection process for the study’s variables of interest will not prolong emergency care or diminish its quality.

Sociodemographic, medical, and clinical data will be analyzed in conjunction with climate data, for example, O_3_ concentrations and air pollution data (Table 1). Data come from MétéoSuisse, Switzerland’s official meteorological service, under the Federal Office of Meteorology and Climatology, and from RESIVAL, the Valais network collecting information on local climatic parameters.

**Table 1:** Databases and variables of interest for meteorological data.

Databases	Variables of interest								
	T_max_	T_min_	T_mean_	NO_2_^a^	PM_25_^b^	PM_10_	SO_2_^c^	O_3_^d^	Pollen
MétéoSuisse [27]	✓	✓	✓						✓
RESIVAL [28]			✓	✓	✓	✓		✓	

^a^NO_2_: nitrogen dioxide.

^b^PM: particulate matter.

^c^SO_2_: sulfur dioxide.

^d^O_3_: tropospheric ozone.

#### Recruitment Procedure in Triage

**Figure 2:**
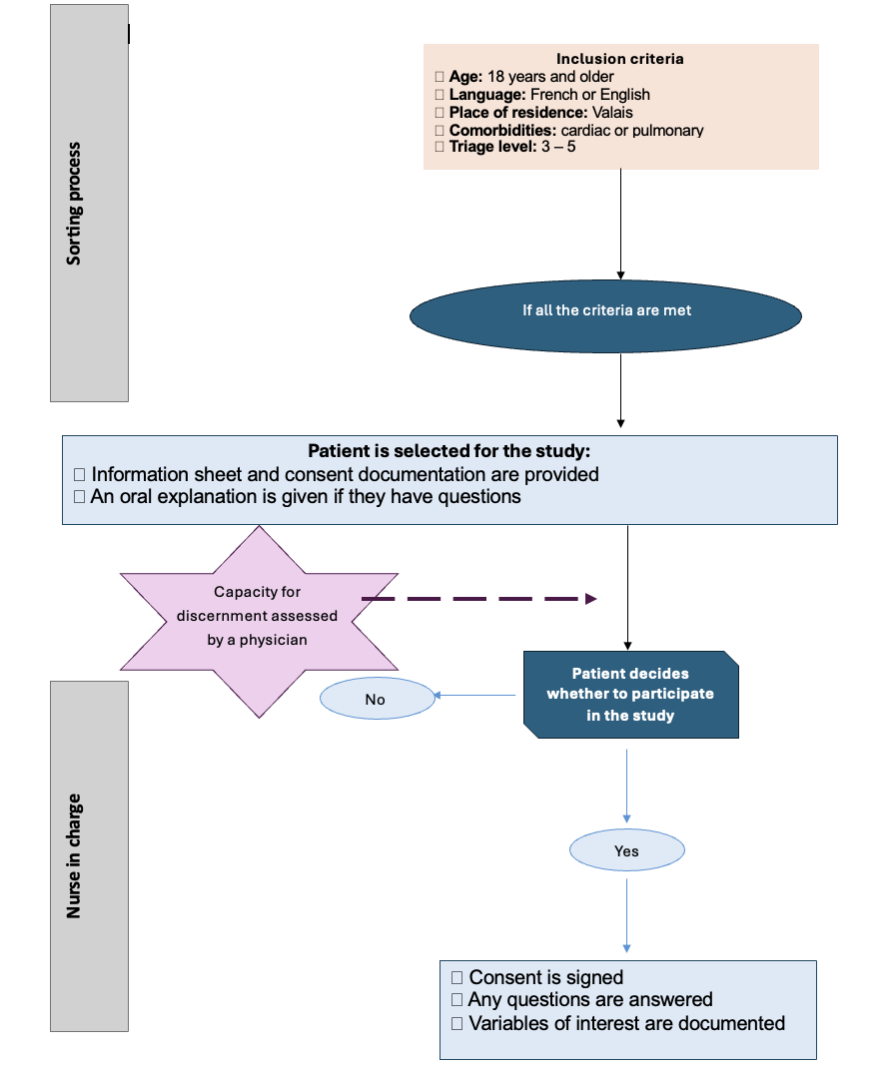
Patient selection algorithm.

The procedures for participant recruitment will be carried out by triage nurses—the first nursing staff patients meet when they arrive at the ED—and the nurse in charge. The principal investigator will explain the participant selection process to all the ED nurses at 2 team meetings (each 45 minutes long at the ED) and through an email containing a Microsoft PowerPoint presentation with a voiceover that will be sent out before those meetings. This will enable nurses to ask pertinent questions about their understanding of the recruitment algorithm. The triage nurses’ role is to prioritize the patient’s health status for their ED stay. They will be responsible for identifying whether patients meet all the inclusion criteria and, thus, for initiating the selection process. The nurse in charge or the triage nurse will then ask the patient whether they consent to participate in the study. If they agree, the nurses will collect the signed consent form. If the patient wishes, they can be given a 24-hour period to reflect. In this case, a stamped addressed envelope with the consent and information sheet will be given to them. As a reminder of the process and to ensure that it is carried out homogeneously, the participant selection algorithm (Figure 2) will be posted in every triage station and at the door of each cubicle. Indeed, nursing care in emergency contexts is often represented using an algorithm, so it is a visual tool that professionals in this field are familiar with. This will promote adhesion to and understanding of the algorithm.

The triage nurse or the nurse in charge will explain the study’s purpose and the patient’s contribution. They will then provide the patient with the study information document and the consent form. These additional explanations will not significantly lengthen triage time nor compromise the quality of care. The patient will have time to read the documents before entering the cubicle. When patients are given their discharge papers home (eg, a prescription, a follow-up appointment, a potential sick note for their employer, or other recommendations), the nurse in charge and the charge nurse, who oversees the operations of their specific nursing unit during a set period while working alongside the team, will ascertain the patient's decision to participate in the study. If the patient agrees, they will collect the signed consent and the document with the variables of interest. Their primary role is to ensure that all nursing functions within the department run smoothly and efficiently, verify whether they have decided to participate in the study, and collect their signed consent form if this is the case. They will answer any patient questions and remind them that their data will be coded and that they may receive a telephone call from the principal investigator.

#### Measuring Temperatures and Pollution

In Valais, the RESIVAL measurement station network monitors and analyzes air pollution levels and calculates mean daily air quality parameters (24 hours from 0 Coordinated Universal Time to 0 Coordinated Universal Time) all across the canton [28]. Daily temperature variables (T_min_, T_max_, and T_mean_) and pollen variables will be taken from MétéoSuisse, Switzerland’s official federal national weather and climate service that operates under the Swiss Federal Department of Home Affairs. It is responsible for weather forecasting, climate monitoring, meteorological research, data collection, and public weather-related services [27].

### Qualitative Phase

#### Overview

The qualitative phase’s primary outcome is to explore patients’ knowledge about climate change and its potential links with their ED visits. The secondary outcome is an exploration of patients’ knowledge about climate change. Indeed, factors outside of medical care, such as health beliefs, knowledge, attitudes, and behaviors such as smoking, are categorized as social determinants of health [29].

The inductive approach will guide the qualitative phase. A thematic analysis will be carried out independently by 2 researchers (OPS and PJPA). The level of theme identification will be latent or interpretative. During the pooling, disagreements will be settled by the intervention of the third researcher (HV).

#### Criteria and Data Collection

A nonprobability sample of patients participating in the study’s quantitative phase will be selected for inclusion in the qualitative phase. The exclusion criterion established is the patient’s incapacity for discernment, according to the patient’s family doctor or family, or having a severe psychiatric disorder. The qualitative phase will be documented through individual interviews of 30-45 minutes until data saturation is reached. Data saturation will be reached when the data collected no longer yields any new significant or thematic information relevant to answering the research question. The theoretical number of interviews required is approximately 10-15. At least 6 weeks after the ED consultation, the principal investigator will contact participants on an aleatory basis by telephone to ask them to participate in a 30-45-minute interview. Six weeks were chosen so as to be far enough removed from any potential hospitalization that followed the index ED consultation. More information will also be collected and explored regarding the social determinants of health, categorized into five domains: (1) neighborhood and environment (where patients live, work, and play), (2) health and quality of access to health care (patients’ use of health services to achieve the best health outcomes), (3) the social and community context (people with whom patients communicate and connect), (4) patients’ access to education, and (5) economic stability (patients’ financial resources). These domains can affect wellness, illness, and disease conditions [30]. By recognizing how social determinants impact health outcomes, nurses can play a key role in addressing health disparities and promoting health equity. Indeed, health disparities—variations in health linked to social, economic, and environmental disadvantages within a society [31], are pressing issues that demand immediate attention. Each area will be explored through a question and a follow-up question. Thus, the semistructured interview guide (Multimedia Appendix 1) consists of 5 questions and 5 follow-up questions. The aim is for the patient to share information about their daily life in relation to these 5 areas.

#### Withdrawal and Discontinuation

In the event of the patient’s withdrawal from the study’s quantitative phase, their data will not be included in the analysis. However, if the patient withdraws from the qualitative phase, their data will still be incorporated into the analysis of the quantitative phase. Participants withdrawing from the qualitative phase will be replaced in order to maintain the estimated number of interviews required. Participants can withdraw from the qualitative phase at any time, which could be due to a change of mind or to any complication resulting in a loss in their capacity for discernment or ability to understand and speak French (eg, an altered state of consciousness, intubation, tracheostomy, and tracheotomy).

### Statistical Analysis

Statistics calculated in a pilot study are used to assess the feasibility of the full-size study and refine its methodology. A variety of methods can be used to address the objectives established for a pilot study, and these need not be statistical [23,26]. Indeed, statistical uncertainty must be taken into consideration before any of the pilot study’s findings are generalized. The goal of publishing results from pilot studies is not to focus on their statistically significant findings but rather to provide their estimated effects on all the measures of interest and to describe the lessons that have been learned and will be informative in planning subsequent studies [23,32].

Factors such as the population’s sociodemographic characteristics, economic conditions, and access to outpatient and hospital care facilities may confound the relationship between climate variables and hospital admissions.

More specifically, numerical and qualitative data will be analyzed according to good clinical research practices. Statistics will be generated from the raw data collected from the ED’s patient records and the appropriate meteorological and air pollution websites (MétéoSuisse and RESIVAL). A database gathering sociodemographic variables (sex, age, place of residence, and marital status), health variables (triage level, reason for the consultation, diagnosis based on the *ICD-10* classification, medical or surgical history, smoking status, and ED readmissions in the last 6 months), meteorological (maximum and minimum temperature), and air pollution data (NO_2,_ PM_2.5_, PM_10_, SO_2_, O_3_, and pollen concentrations) will be prepared on a Microsoft Excel spreadsheet. They will be imported into and analyzed using SPSS software (version 29.0; IBM Corp). Descriptive statistics such as mean and SD (for quantitative variables) and frequency and percentages (for qualitative variables) will be calculated. Parametric statistical tests will be applied to normally distributed variables. Nonparametric statistical tests will be used for variables with non-Gaussian distributions. This data analysis will enable us to better describe the study participants’ profiles. To estimate the effects of meteorological, sociodemographic, and health data, we will calculate a conditional quasi-Poisson regression and distributed lag nonlinear models. The model will estimate the association between hospital admissions and predictor variables (sociodemographic, health, meteorological, and air pollution variables). One advantage of the Poisson pseudomaximum likelihood estimator is that the scale of the dependent variable does not affect the parameter estimates [10]. In this type of regression model, a pseudolikelihood is applied to properly scale the SD of the coefficients proportionally to the potential overdispersion. We will use overdispersed generalized additive models with random effect meta-analysis to investigate the associations between variables [8]. ANCOVA models will also be developed to compare the means of a continuous dependent variable (hospital admissions) across multiple factor variables (autumn, winter, spring, and summer) and to determine covariates’ effects (sociodemographic, health, meteorological, and air pollution variables). This approach ensures a more nuanced understanding of how various factors and covariates collectively influence hospital admissions. Finally, the participant inclusion rate and study retention or drop-out rates will also be estimated, as will the total sample size required for the full-scale study.

The statistical significance threshold will be set according to the number of variables and the size of the database developed (2-sided *P* values<.01 will be considered statistically significant, with 95% CIs and Bonferroni adjustments for multiple comparisons).

### Feasibility and Acceptability Phase

Feasibility refers to the practicality and appropriateness of the processes involved in patient selection and data collection within the pilot study. It evaluates the ability to recruit and retain participants, the functionality of inclusion and exclusion criteria, the adequacy of recruitment methods, and the efficiency, reliability, and acceptability of data collection tools and procedures [33,34]. Acceptability refers to the extent to which the study recruitment and data collection procedures, and interventions are considered appropriate, satisfactory, and appealing to participants, and other stakeholders. It evaluates the willingness of participants and involved personnel to engage with and adhere to the study’s requirements [33,34]. The acceptability will be explored through an inductive approach. Three focus groups, each consisting of 4-6 ED nurses, will be conducted to explore variables such as time, objectives of the pilot study, implications, satisfaction, potential barriers, and facilitators to collect data, and ease of the process through open-ended questions. Finally, patients’ level of acceptability of the interview guide will be explored through an open-ended question at the end of the individual interview “Do you feel that the guide enabled you to share meaningful insights?”

Feasibility will be assessed through 2 different approaches. The first approach will assess feasibility through the same focus groups used to assess acceptability. On a scale of 1=”don’t agree at all” to 5=”completely agree,” nurses will rate the process of the communication and training process to which they have been subjected (study protocol, mentoring, printed selection algorithm, principal investigator’s feedback, and communication about the project). The second approach will consist of the proportion of expected data and data collected.

### Ethical Considerations

The research protocol (2024-00900) for this pilot project was presented to its different partners. A request for authorization to proceed was presented to Swiss Ethics. The cantonal ethical commission for research on human beings, represented by Jean-Marie Annoni, gave its final approval on October 23, 2024. The support of the University of Applied Sciences and Arts Western Switzerland is assured. Participation in the study will be voluntary and pose no risk to the patients. In the context of this study, it is highly unlikely that participants will be exposed to any inconvenience or risk. Whether they participate in the study will in no way alter their care pathway or the quality of the care and follow-up they receive. Should the participant request them, the principal investigator will send them the pilot study’s results by email or post. Participation in this study will be of no direct benefit to the patient. However, the results may help improve the overall delivery of care by providing emergency medicine that is better adapted to the patient’s health status and needs. Finally, the preliminary results of the development and usability pilot study will enable the research team to propose a larger-scale study.

Given the nature of the variables to be collected, personal data will be coded to protect the life, health, dignity, integrity, right to self-determination, privacy, and confidentiality of the people participating in the research. The coding process will be based on the best-practice recommendations found in Article 29 of the Working Group on Data Protection (the independent European advisory body on data protection and privacy). A collaboration agreement has been drawn up with Hôpital du Valais in the canton’s French-speaking area. The principal investigator will be granted access to data obtained with patient consent for the duration of the study. The data collection process will be carried out in compliance with the appropriate data protection, human research, and ethical principles, thus ensuring respect for patients’ rights and the confidentiality of their medical information. The coding key will be kept separate from the study data.

For the purposes of this development and usability pilot study, data will be stored on the Haute École de Santé Valais-Wallis’ OneDrive server. The coding key between personal data and the numerical code, as well as the transcripts of each individual interview, will be kept by the principal investigator.

## Results

Data collection started on September 21, 2024, following approval by the ethical commission. Data collection will take place over 1 year, until September 20, 2025. After 1 season of data collection, that is, between September 21, 2024, and December 21, 2024, a total of 60 patients were screened. The quantitative phase will begin in February 2025.

## Discussion

### Principal Results

The results of this development and usability pilot study will enable us to assess the feasibility of its methods and procedures with a view to carrying out a larger study. Indeed, it is important to adapt the patient selection algorithm to the clinical field’s requirements and frontline carers’ needs. By definition, the flow of patients coming to EDs cannot be predicted. The selection algorithm must be simple and not significantly delay a patient’s treatment or slow its commencement. Our assumption is that we will have to make changes in 2 stages. These will still be communicated to managers and the entire care team. Concerning sociodemographic profiles, the hypothesis is that cardiovascular disease, active smoking, previous smoking, and diabetes are factors of vulnerability to climate change. In conjunction with the qualitative part, we hypothesize that vulnerable patients are aware of reduced quality of life during extreme temperatures or pollution peaks. However, little, if any, action is taken to anticipate and prevent this. Patients’ levels of technical literacy are, therefore, potentially low. Finally, we hypothesize that the qualitative interview guide’s acceptability level is high. The interview guide has been pretested, and the exchange lasts no more than 45 minutes [32].

The results of the present development and usability pilot study could constitute a first step toward developing sustainable, ecological care. Indeed, today’s duality of a health care system that provides benefits to patients and the population but has deleterious environmental consequences is becoming less and less acceptable. Finally, this development and usability pilot study will establish guidelines for a future larger-sized study.

### Limitations

Data will be collected over 1 year so that all 4 seasons and their particularities—especially regarding climatic variables—can be studied. This development and usability pilot study is a single-center study and will only represent results from the Hôpital du Valais’ ED in Sion. Finally, its objective is to assess and analyze the potential for adult patient recruitment, the reasons for patients’ consultations, and their level of triage. It will also test the acceptability of an interview guide to explore patients’ knowledge of climate change and its links with their ED visits. These preliminary results will constitute the first milestone in the assessment of the practicality of recruiting and retaining participants and in the determination of the most appropriate participant selection process to ensure stakeholder adherence for a full-scale study.

### Conclusions

Climate change is a central concern for the discipline of nursing. Nurses are strategically placed to respond to the impacts of climate change through their practice, research, and training in developing, implementing, and sustaining innovation for climate change mitigation and adaptation. To do so, they must be given the tools to come to grips with the global health emergency, that is climate change. Taking stock of the current situation, particularly in the alpine canton of Valais, in Switzerland, will require a thorough assessment. This development and usability pilot study’s preliminary results will be used to inform a future large-scale study whose findings we may be able to generalize and identify climate change’s impact on the environment and the health of Valais’ population.

## Appendix

Multimedia Appendix 1 Interview guide.

## Abbreviations

ED: emergency department
ICD-10: International Statistical Classification of Disease, Tenth Revision
PM: particulate matter
NO2: nitrogen dioxide
O3: tropospheric ozone
SO2: sulfur dioxide
SPIRIT: Standard Protocol Items: Recommendations for Interventional Trials

## Data Availability

All data generated or analyzed during this study are included in this published article.
