# Supplementary material for: Exploring Climate Change’s Impact on the Cardiopulmonary Health of Adults Living in the Canton of Valais, Switzerland: Protocol for a Development and Usability Pilot Study
Source: JMIR Res Protoc. 2025 Mar 25;14:e67128. doi: 10.2196/67128 (PMC11979528; doi:10.2196/67128)
Supplement: Multimedia Appendix 1 [file resprot_v14i1e67128_app1.pdf]

(i) Perceptions, experiences and feelings about climate change

1. How do you think climate change might affect your health and emergency room visits, or those of your loved ones?

2. Have you noticed any changes in your health or that of your loved ones that could be linked to climate change?

Could you elaborate on your point of view?

3. Does climate change affect your daily habits?

Could you tell us more about this?

(ii) Neighborhood and environment, and (iii) Social and community context

4. Can you tell me a little about your neighborhood and the environment in which you live?

Can you tell me more about your relationships with your neighbors?

5. What aspects of your environment could influence (positively or negatively) your health and well-being?

Can you give me an example?

(iv) Health and access to healthcare

6. Are there any difficulties you have encountered in accessing healthcare?

Can you give me some examples?

(v) access to education, and (vi) economic stability

7. Can you tell me about your education and how it led you to your current job?

What fields of study or training have you followed, and how have they influenced your career choice?

8. How do you feel about your finances and your ability to support yourself?

Can you tell me more?

9. Are there any challenges you face or factors that influence your sense of economic security?

Do you have anything to add?
